# Anticancer Drugs Paclitaxel, Carboplatin, Doxorubicin, and Cyclophosphamide Alter the Biophysical Characteristics of Red Blood Cells, In Vitro

**DOI:** 10.3390/biology12020230

**Published:** 2023-01-31

**Authors:** Elisaveta Skverchinskaya, Nadezhda Levdarovich, Alexander Ivanov, Igor Mindukshev, Anton Bukatin

**Affiliations:** 1 Sechenov Institute of Evolutionary Physiology and Biochemistry of the Russian Academy of Sciences, 194223 Saint-Petersburg, Russia; 2 Laboratory of Renewable Energy Sources, Alferov University, 194021 Saint-Petersburg, Russia; 3 Institute for Analytical Instrumentation of the Russian Academy of Sciences, 198095 Saint-Petersburg, Russia

**Keywords:** red blood cells, chemotherapy, anemia, microcirculation, laser diffraction, osmotic fragility test, microfluidics

## Abstract

**Simple Summary:**

An important issue in cancer chemotherapy is minimizing its side effects. The extreme toxicity of chemotherapy drugs is due to their task of preventing the multiplication of cancer cells and causing cancer cell death. One of their most common undesirable side effects is anemia, which is caused by a decrease in the number of red blood cells (RBCs) circulating in blood, which in turn results in a lack of oxygen in tissues. The manifestation of anemia is associated not only with the inhibition of the hematopoietic function of bone marrow but also with direct damage to RBCs during the drugs’ infusion and circulation. Here, we investigated how frequently used chemotherapy drugs directly affect RBCs. Our results show that chemotherapeutic drugs, whose main task is to damage the DNA of cancer cells and prevent their division, have a noticeable toxic effect on RBCs. However, this effect is lower than the effect caused by drugs, which disrupt the dynamics of the cytoskeleton during cell division. Direct simulation of RBCs’ transport in microchannels of a microfluidic device was allowed to integrally assess the cells’ functionality and the capability of passing through microcapillaries where gas transport mainly occurs. We demonstrate that after exposure to drugs, regardless of their type, the number of damaged cells did not exceed 10%, which indicates the balance of the drugs’ therapeutic doses. Our data along with the developed research method could be used to work out an effective combination of chemotherapeutic drugs as well as to calculate the efficient therapeutic drug doses for cancer treatment to reduce anemia side effects.

**Abstract:**

Red blood cells (RBCs) are the most numerous cells in the body and perform gas exchange between all tissues. During the infusion of cancer chemotherapeutic (CT) agents, blood cells are the first ones to encounter aggressive cytostatics. Erythrocyte dysfunction caused by direct cytotoxic damage might be a part of the problem of chemotherapy-induced anemia—one of the most frequent side effects. The aim of the current study is to evaluate the functional status of RBCs exposed to mono and combinations of widely used commercial pharmaceutical CT drugs with different action mechanisms: paclitaxel, carboplatin, cyclophosphamide, and doxorubicin, in vitro. Using laser diffraction, flow cytometry, and confocal microscopy, we show that paclitaxel, having a directed effect on cytoskeleton proteins, by itself and in combination with carboplatin, caused the most marked abnormalities—loss of control of volume regulation, resistance to osmotic load, and stomatocytosis. Direct simulations of RBCs’ microcirculation in microfluidic channels showed both the appearance of a subpopulation of cells with impaired velocity (slow damaged cells) and an increased number of cases of occlusions. In contrast to paclitaxel, such drugs as carboplatin, cyclophosphamide, and doxorubicin, whose main target in cancer cells is DNA, showed significantly less cytotoxicity to erythrocytes in short-term exposure. However, the combination of drugs had an additive effect. While the obtained results should be confirmed in in vivo models, one can envisioned that such data could be used for minimizing anemia side effects during cancer chemotherapy.

## 1. Introduction

According to estimates from the World Health Organization, cancer is the first/second leading cause of death [1]. Cancer treatment is a multi-component process in which, along with immunotherapy, surgery, and radiotherapy, chemotherapy still occupies a key role [2]. Chemotherapy (CT) is a powerful and aggressive treatment for cancer patients, the main goal of which is to destroy cancer cells. They are highly proliferative cells; therefore, the main mechanisms of CT drugs’ action include DNA strand bonding and blocking DNA replication, blocking of metabolic pathways, and preventing replication. Additionally, for chemotherapeutic drugs’ action, cell membranes represent either a possible target or an obstacle to the drugs’ effect—suppression of malignization.

Currently, the main CT regimens for solid tumours include anthracyclines and taxanes accompanied by alkylating agents. The therapeutic effect of taxanes (paclitaxel (TAX), docetaxel) is due to blocking the depolymerization of microtubule tubulin, which leads to the inhibition of cell division [3]. The antiproliferative effect of platinum-based drugs (carboplatin (PLAT), cisplatin, oxaliplatin, etc.) is DNA–DNA and DNA–protein crosslinking, which leads to blockage of DNA replication and/or reparation [4]. These drugs work similarly to alkylating antineoplastic agents (anthracyclines), such as doxorubicin (RUBI). These agents’ actions include interaction with DNA by intercalation, inhibition of protein kinases, and stabilization of the topoisomerase IIα complex after DNA cleavage, which in turn stops the replication process [5]. An exception to anthracycline drugs is cyclophosphamide (PHOS), whose action is mainly due to its metabolite phosphoramide mustard. Along with immunomodulation, this metabolite performs DNA alkylation, which causes cytotoxic apoptosis of tumour cells [6].

Although circulating erythrocytes (red blood cells, RBCs) have never been a target for cancer chemotherapy, they are the first to encounter aggressive cytostatics during infusion of CT drugs. RBCs lack a nucleus; therefore, the effect of CT drugs may be primarily due to the interaction with the lipid part of cell membrane and with the cytoskeleton. It has been shown that anthracyclines inhibit actin polymerization [6], which may play an important role in reducing the mechanical strength of erythrocytes and have a significant effect on reducing their rigidity. Moreover, interaction of anthracyclines only with the cell membrane without penetration into cells is sufficient for cancer cell death [7]. Thus, the target of action of anthracyclines can be both the lipid membrane and the cytoskeleton of erythrocytes. Another target in RBCs was reported to be the inhibition of Na-K-ATPase activity, which leads to volume regulation disruption [8].

Paclitaxel is another widely used chemotherapy drug from the class of taxanes. Its antiproliferative effect is linked to the ability to block the depolymerisation of tubulin and, thus, to stabilise microtubules. Mature erythrocytes lack microtubules but, as was shown by proteomic analysis, possess tubulin [9]. The treatment of erythrocytes with another taxane, Taxol^®^, resulted in an increase in the membrane tubulin pool and a decrease in erythrocyte deformability [10]. Additionally, it has been established that paclitaxel in erythrocyte membranes can also act on actin, a component of the cytoskeleton structure [11]. It has been shown that paclitaxel induces ionic pores in planar lipid bilayers. The drug molecules bind randomly on the cell surface in the form of single particles or clusters, and the favourable energetics in the adsorbed drug clusters can lead to the creation of ion channels [12,13].

Platinum drugs are actively used in combination therapy for solid tumours [14,15]. It is believed that with respect to erythrocytes, the main negative effect of platinum drugs is due to a decrease in antioxidant protection [16]. However, not all platinum preparations have equal prooxidative capabilities [17]. For example, treatment of erythrocytes with cisplatin leads to their transformation into stomatocytes [18] and to the disruption of lipid asymmetry with release of phosphatidylserine to the outer surface, which in turn increases procoagulant potential [19].

Another widely used CT drug is cyclophosphamide, which is a synthetic alkylating cytostatic used as an antitumour and immunosuppressive agent [20]. Cyclophosphamide must be metabolised to form mustard phosphoramide in order to exert its antitumour effect [21]. Little is known about the direct effect of cyclophosphamide on erythrocytes. In in vitro experiments, cyclophosphamide was shown to reduce erythrocyte antioxidant protection through a decrease in glutathione levels and the activity of the following enzymes: glutathione-S-transferase, catalase, glutathione peroxidase, and glutathione reductase [22].

In summary, different CT drugs might have different action on RBCs, but anemia during cancer treatment is the major multifactorial side effect, which occurs in 30–90% of patients with solid tumors (breast, lung, colon/rectum, stomach, and ovarian cancer) [23]. It can be induced not only by the drug itself but also by its toxic solvent used to improve solubility in case of taxanes [24,25]. In addition to reduced quality of life, anemia contributes to a suboptimal response to treatment, both through disruption of the CT dosing regimen and through limitation of the cytotoxic effect of CT in the setting of tumor hypoxia [26]. Two main pathophysiological causes of anemia in chemotherapy of oncology are: decreased RBCs formation due to the suppression of hematopoietic organs and increased RBC destruction in the circulation due to the direct cytotoxic effects of CT drugs and the release of substandard RBCs into the bloodstream [27].

Standard laboratory methods to determine the condition of RBCs are hematology analysis, which assesses their mean corpuscular volume, hemoglobin concentration, red cells distribution width, etc.; an osmotic fragility test, which indirectly characterizes the RBC’s deformability; different flow cytometry tests, which show membrane structure and composition, enzyme activity, etc.; spectrophotometry for calculation of the percentage of hemolyzed cells and hemoglobin species; and confocal microscopy, which evaluates a cell’s morphology. The limitation of all these methods is that they can only indirectly predict changes in RBCs’ functionality. In contrast, modern microfluidic technologies can be used to simulate microcirculation conditions, study microrheology, and assess erythrocyte behavior in fluid flow on a single cell level. Thus, it is possible to uncover the relationship between changes in the RBC membrane and the dynamics of microvasculature flow caused by these alterations [28].

Analysis of erythrocyte behavior in microfluidic devices can directly detect microcirculatory disorders [29], the duration of blood bank storage [30], and oxidative stress level [31]. In the field of oncology, microfluidics is a key technique for liquid biopsy and has been successfully used for label-free sorting and isolation of circulating tumour cells [32] and extracellular vesicles [33].

However, little is known about the direct influence of CT drugs on the microcirculation of RBCs, which might be one of the significant reasons for anemia during cancer chemotherapy. We could not find any investigation of the simulation of RBC behavior under the microcirculation conditions in microfluidic devices after exposure to CT drugs. Moreover, current treatment protocols use a combination of two or more drugs to enhance their antiproliferative activity [34]. Furthermore, there is even less data on the combined effect of several CT drugs on red blood cells. From this perspective, knowledge of the off-target effects of CT drugs is crucial to understand possible further erythrocyte transformation and to make informed clinical decisions regarding drug selection and treatment regimen adjustments.

The aim of this study is to provide insight into the transformation of erythrocytes during cytotoxic injury caused by commercial pharmaceutical CT drugs in the first hours after exposure and to evaluate the contribution of cytological abnormalities to the biomechanical parameters of erythrocytes. To this end, we have investigated how the cytological and biophysical parameters of RBCs are changed when the cells are subjected to basic chemotherapy drugs with different action mechanisms. For this task, we have used flow cytometry analysis to determine cell viability and asymmetry of lipids in the cell membrane; small-angle light diffraction to figure out the cell shape and osmotic fragility; and confocal microscopy to analyse morphology. These methods were combined with microfluidic simulations of microcirculation in microcapillaries to complexly define changes in RBCs’ biophysical characteristics. We expect that a developed in vitro testing comparative approach of the drugs’ direct influence on erythrocytes will provide an opportunity to assess the cells’ resistance to a peak drug load during the infusion in the first hours. This approach can additionally facilitate the optimization of drug loading and therapeutic dose in order to reduce chemotherapy side effects.

## 2. Materials and Methods

### 2.1. Preparation of RBC’s Suspensions and Their Treatment

Volunteers’ blood was collected by venipuncture of the anterior cubital vein into S-monovette tubes (9NC, Sarstedt, Nümbrecht, Germany) with the addition of 2 mM EGTA. All volunteers were healthy at the time of blood sampling and did not take any medication for more than two weeks before blood donation. Each participant signed an informed consent for blood sampling and the anonymous presentation of the results.

The basic HEPES buffer was isotonic and was prepared according to the protocols for clinically approved LORCA analyzer (Laser-assisted Optical Rotational Cell Analyzer, https://lorrca.com/, accessed on 1 January 2020) [35]. It had the following composition: 10 mM HEPES, 140 mM NaCl, 5 mM KCl, 2 mM MgCl_2_, 5 mM D-glucose, 2 mM EGTA, pH = 7.4 (pH-meter Metler Toledo, Columbus, OH, USA), and 300 mOsmol/kg H_2_O (mOsmol) controlled by cryoscopic osmometer Osmomat 3000 (Gonotec, Germany).

RBCs’ suspensions were obtained by whole blood centrifugation at 400 g for 3 min (Centrifuge ELMI-50CM, Elmi, Latvia) and two subsequent washes in HEPES buffer using the same centrifugation parameters. Washed RBCs were resuspended in HEPES buffer to a concentration of 5 × 10^8^ cells/mL (corresponding to hematocrit 4–4.5%) to ensure that the specific concentration of drugs from different donors was similar. The hematological parameters of the blood and RBC suspension were controlled by the hematological counter Medonic-M20 (Boule Medical A.B., P.O. Box 42056 SE-126 13, Stockcholm, Sweden).

The suspensions of RBCs were incubated with commercial pharmaceutical CT drugs at 37 °C for 3 h: paclitaxel—Paclitaxel-Ebewe (Sandoz, Ebewe Pharma Ges.m.b.h.Nfg.KG A-4866 Unterach, Austria), carboplatin—Carboplatin-Teva (Pharma B.V., Pharmachemie B.V., Swensweg 5, P.O. Box 552, 2003 RN Haarlem The Netherlands), doxorubicin—Farmorubicin^®^ (Pfizer, Western Australia - 6102, Australia), cyclophosphamide—Endoxan^®^ (Baxter, Halle/Westfalen, Germany), and their combinations TAX_PLAT and RUBI_PHOS, which are widely used in CT practice. All concentrations of used CT drugs corresponded to the maximum recommended therapeutic doses: TAX 175 mg/m^2^, PLAT 400 mg/m^2^, PHOS 600 mg/m^2^, and RUBI 150 mg/m^2^. Given that the drugs act on a finite number of cells, we calculated their concentrations for RBC concentration 5 × 10^8^ cells/mL. The final TAX concentration was 17.5 µg/mL, PLAT—32.5 µg/mL, PHOS—65 µg/mL, and RUBI—16.5 µg/mL. The CT drugs were prepared prior to use, following the manufacturer’s recommendations. The concentration of each drug was calculated according to the statistical average body surface area S = 1.81 m^2^ and an average circulating blood volume V = 4.5 L.

### 2.2. Osmotic Fragility Test

The degree of osmotic fragility is used as a surrogate sign of deformability disorders [36] because it is a composite indicator of RBC shape, hydration, and, within certain limits, susceptibility to fracture in vivo [37]. The osmotic fragility test (OFT) is based on laser diffraction at small scattering angles (0–12°) performed by a laser particle analyser “LaSca-TM” (LLC “BioMedSystem”, Saint-Petersburg, Russia). This method is much quicker than the standard OFT [37] and provides an osmotic resistance curve, which is the number of lysed cells in the buffer with different osmolality. It is sensitive to cytological changes in cells and can be applied to different cell suspensions including RBCs [37,38]. The analyzer was calibrated using 3, 6, and 10 µm latex beads (Invitrogen, Molecular Probes, Eugene, OR, USA).

The procedure of OFT was as follows: we placed the treated or control RBCs (1 × 10^6^ cells/mL) in the cuvette in HEPES buffer (300 mOsmol). During the registration of the scattered laser intensity (SLI), we manually changed the buffer osmolality in the range of 300–100 mOsmol adding to the sample aliquots of dH_2_O together with additional RBCs to maintain their concentration constant. The cuvette with the sample was temperature-controlled at 37 °C and equipped with a magnetic microstirrer (1200 rpm), which ensured rapid mixing of the suspension. The intensity of scattered light from the RBCs was continuously detected by forward scattering. The laser diffraction at the selected angles of detection made it possible to register cell swelling (increase in scattered light intensity at 1–6° angles) and hemolysis (decrease in scattered light intensity at 1–12°) [39].

The following parameters of RBCs were evaluated:

1. H50, mOsmol—the buffer osmolality, at which 50% of cells were lysed.
2. W, mOsmol—the distribution width of the osmotic resistance curve, at which 90% and 10% lysis occurs (W = H90–H10, mOsmol). It is a characteristic of the heterogeneity of the RBCs’ pool.
3. MCV_osm_, fL—the hydrodynamic cell volume versus the buffer osmolality. The MCV_osm_ curve was normalised to the MCV value at 300 mOsmol measured by the hematological analyser.
4. The asphericity index, AI, %, which is the normalised amplitude of the SLI oscillations of the RBCs in the buffer with physiological osmolality (300 mOsmol). This index is proportional to the shape asymmetry of the cells and can be used to distinguish the normal discoid and spherical shape of RBCs.

### 2.3. Flow Cytometry Analysis

Flow cytometry analysis was performed on CytoFLEX (BeckmanCoulter, Brea, CA, USA), with analysis of 20,000 events. For RBC detection, we used the forward scattering and side scattering coordinates (FSC/SSC), which gave information about cell size and structure.

Calcein-acetoxymethyl ester (Calcein-AM, C-AM; Molecular probes, Eugene, Oregon, USA) was used to estimate cells’ esterase activity. After the incubation of RBCs with anticancer drugs, the suspension was diluted to 5 × 10^6^ cells/mL and stained with Calcein-AM (5 μM, 40 min, 37 °C) in 300 µL of HEPES buffer. Mean Fluorescence Intensity (MFI) values of the control cells were taken as 100% efficiency of intracellular esterases in each individual experiment. To obtain intracellular esterase activity data of treated RBCs, the MFI values were normalised to the MFI of the control.

Transformation of the cytoskeleton with the formation of band3 protein clusters characterizes the final stages of RBC life [40]. This transformation of the cytoskeleton by CT drug action was assessed using the eosin-5-maleimide test (EMA; Molecular probes (Eugene, Oregon, USA). We incubated RBCs (5 × 10^6^ cells/mL) with 0.07 mM EMA in HEPES buffer for 40 min, 25 °C.

Lipid asymmetry was assessed by the externalization of phosphatidylserine (PS) to the outer side of the membrane by the Annexin V test. RBCs (5 × 10^6^ cells/mL) were incubated with Annexin V-FITC (Biolegend, Amsterdam, The Netherlands) for 15 min, 25 °C, in HEPES buffer. EGTA in HEPES buffer was replaced by 2 mM Ca^2+^. The concentration of Annexin V-FITC was taken according to the manufacturer’s recommendations.

In all the experiments, the fluorescent intensity was registered in FITC-channel (excitation 490 nm, emission 530 nm). For all CT drugs, the intensity of the fluorescent signal of the negative control (*n* = 3) was checked. To correctly record the signal of RBCs exposed to RUBI, which has its own laser-induced fluorescence, the negative control (RUBI-treated RBCs without dye staining) was subtracted. For RUBI, MFI did not exceed 0.5–1.1% of the total intensity of control cells stained with C-AM or EMA, which is negligible. Furthermore, it did not give any signal in the gate of Annexin V-positive cells (Annexin+, Ann+). For other CT drugs, the MFI level of the negative control was even lower.

### 2.4. Confocal Microscopy

An inverted Leica TCS SP5 MP confocal laser microscope (Leica Microsystems *GmbH, Wetzlar,* Germany) was used to visualize the RBCs’ morphology after incubation with the anticancer drugs. We diluted 10 μL of treated RBCs in 200 μL of HEPES buffer with 3.7% of bovine serum albumin to prevent echinocytosis and placed this sample in Petri dishes (35 mm) with a centre hole replaced by a coverslip (SPL Lifesciences, Pocheon South, Korea). The microphotographs were processed using ImageJ software (Public Domain). 

### 2.5. Microfluidic Analysis

For an integrative assessment of changes in RBCs under the influence of CT drugs, we evaluated RBCs’ transit velocities through 2.5 × 8 × 200 µm microchannels in custom-made microfluidic chips [31,41]. This is an integral method for assessing functional disorders accumulated by erythrocytes, which takes into account changes in their shape, volume, adhesion, elasticity, and deformation characteristics.

The preparation of the microfluidic chips started with fabrication of polydimethylsiloxane (PDMS) replicas. We degassed the mixture of Sylgard 184 Silicone Elastomer Base and the Curing Agent 10:1 (Dow Corning, Midland, Michigan, USA). Then we filled the silicon mold with this mixture and cured it at 65 °C for 4 h. After the curing, we separated the replica from the mold and cut out the inlet and outlet holes with a 1 mm biopsy puncher. Then the PDMS replica was covalently bonded with a 75 × 25 × 1 mm glass slide after oxygen plasma treatment (PINK GmbH Thermosystem, Wertheim, Germany).

Before the experiment, we filled the microchips with HEPES buffer to prevent RBC adhesion to PDMS. All samples (5 × 10^7^ cells/mL) were introduced into the chip under constant hydrostatic pressure. Recording of RBC transit in microchannels was performed via XIMEA MC023MG-SY video camera (XIMEA Corp., Lakewood, CA, USA) with a 400 fps frame rate through a Leica DM4000B LED microscope (Leica Microsystems GmbH, Wetzlar, Germany) with an N PLAN L 20×/0.40 objective (Leica Microsystems, Wetzlar, Germany). For each sample, we recorded at least 4–7 different channels of a 16-channel microchip for obtaining statistically correct data.

Analysis of obtained images was carried out by a custom MATLAB (The MathWorks) script [42], which calculated RBC transit velocities. Then the obtained values were normalised to the average RBC’s velocity in wide channels, which corresponded to the average velocity of the fluid flow. After that, we constructed the probability density functions (Origin 2021, OriginLab Corporation) for each experiment and averaged them over all experiments.

### 2.6. Free Hemoglobin and Hemoglobin Species Calculation

To measure free hemoglobin (Hb) in RBC suspension, incubated samples were centrifuged at 400 g for 3 min. Then an aliquot of supernatant RBCs was diluted 20-fold with dH2O and analysed with a spectrophotometer (SPECS SSP-715-M, Spectroscopic systems LTD, Moscow, Russia). The calculation of the free hemoglobin (Hb) was based on the optical density of the plasma solution and lysate at 540 nm, where the optical density of the whole blood lysate was taken as 100%. For the correct calculations, the absorbance was also assessed at 700 nm to eliminate the influence of the solution’s turbidity. The free Hb% was calculated according to Tarasev [43].

To analyse the formation of Hb forms under the action of CT drugs, the hemolysate of each sample (*n* = 3 donors) was scanned at 560, 577, 630, and 700 nm. The percentage of oxidised Hb was calculated by molar extinction coefficients of Hb species according to the method of Kanias [44].

### 2.7. Statistics

Microfluidic analysis data such as cell velocities and percentage of microchannel occlusions is presented as mean ± SE; the error bars on velocity histograms are shaded. Other data is presented as mean ± SD. To analyse the flow cytometry data, CytExpert (BeckmanCoulter, Inc. Brea, CA 92821, USA) and FCS Express Flow 7 (De Novo Software, Pasadena, USA) were used. To assess the laser diffraction data, the original software of the laser analyser LaSca-TM was used. Statistical significance was evaluated by Excel 16 (Microsoft, Redmond, WA, USA), GraphPad Prism 9 (GraphPad Software, San Diego, CA, USA), and Origin 2021 (OriginLab Corporation).

The differences between the groups were analysed by GraphPad Prism 9. The normal distribution was tested with the D’Agostino and Pearson normality test (in accordance with the recommendations of the GraphPad Prism guidelines). For multiple comparisons, one-way ANOVA followed by Tukey’s multiple comparisons test Tukey HSD post-hoc (passed normality test), or Dunn’s multiple comparisons test (no passed normality test) were used; values of *p* < 0.05 were considered statistically significant. All the obtained data and statistics are presented in Table A1.

## 3. Results

### 3.1. Osmotic Fragility Test Based on Laser Diffractometry

Unlike other blood cells, RBCs placed in a medium with an osmolality below the physiological values of blood plasma (285–305 mOsmol) become swollen. With a further decrease in osmolality, they lyse. Therefore, the OFT can be used as an integral indicator of RBC membrane disruption by CT drugs. OFT allows evaluation of a wide range of cell parameters. The most important is osmotic fragility/rigidity, which is a composite parameter of biophysical and morphological properties of RBCs’ membranes: shape, cytoskeleton rigidity, and hydration. It analyses the tendency of cells to lyse in circulation [45].

#### 3.1.1. OFT: CT Drugs Impair RBCs’ Osmotic Resistance

**Figure 1:**
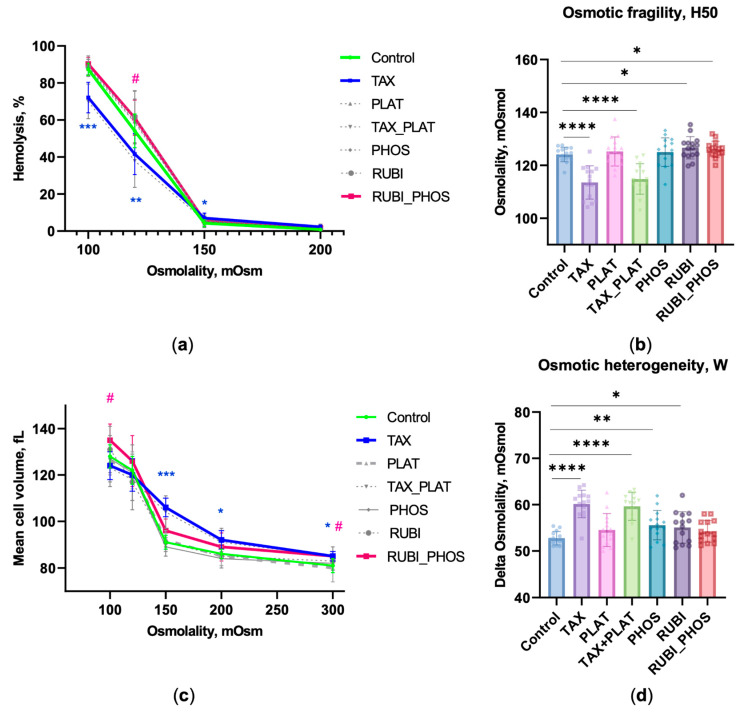
OFT demonstrates that CT drugs alter RBCs’ osmotic resistance. (**a**) The osmotic lysis curves. TAX-treated cells started lysing earlier than the control ones, but were more rigid at 120 and 100 mOsmol; cells incubated with RUBI_PHOS were more sensitive to hemolysis at low osmolality 120 mOsmol; (**b**) H50 is the osmolality of the buffer at which 50% of the RBCs were lysed; CT drugs affect RBCs’ membranes differently: TAX and TAX_PLAT caused increased osmotic stiffness of RBCs, and RUBI_PHOS caused osmotic fragility of RBCs; (**c**) Quantification of MCV during osmotic fragility test: MCV increased for TAX and TAX_PLAT earlier than for other drugs and control cells; (**d**) OFT revealed increased osmotic heterogeneity in the population of RBCs exposed to CT drugs. Data are presented as mean ± SD, *n* = 15 donors, one way ANOVA, Tukey HSD post-hoc; *, *p* ≤ 0.05, **, *p* ≤ 0.01, ***, *p* ≤ 0.001, ****, and *p* ≤ 0.0001 compared to control. Pink #, *p* ≤ 0.05 refers to RUBI_PHOS compared to control, blue asterisks refer to TAX and TAX_PLAT compared to control.

The study of RBCs’ fragility/rigidity after exposure to CT drugs was performed using two methods—the percentage of hemolysis at each buffer osmolality was calculated (Figure 1a), as was the osmotic fragility H50 (Figure 1b). Analysis of the hemolysis curve showed that RBCs under the action of TAX and TAX_PLAT started lysing earlier than cells in the other groups including control cells (Figure 1a). This means that these cells became fragile. Additionally, at low osmolality (100 mOsmol), TAX- and TAX_PLAT-treated cells showed a higher degree of rigidity to hypotonic load. It was also shown that the combined action of RUBI_PHOS increased the osmotic fragility of RBCs at a low osmolality of 120 mOsmol. Additionally, the hemolysis in this case was higher than that of RUBI and PHOS alone (Figure 1a, Table A1).

To assess the fragility/rigidity of RBCs, we used the H50 value, which indirectly represents the deformability of the cells [40,45,46]. The higher the H50 value, the more fragile the cells are. Incubation with TAX for 3–4 h resulted in a decrease of the H50 value by 6.4 ± 1.1 mOsmol, *n* = 13 donors. Incubation of RBCs with TAX_PLAT had the same effect (Figure 1b), but PLAT itself did not cause changes in the resistance of RBCs to osmotic loading.

The combined action of RUBI_PHOS caused a decrease in osmotic resistance (Figure 1a). In this case, lysis of half of the cell pool occurred at osmolality values higher than in their separate action. Thus, the action of TAX and TAX_PLAT contributes to an increase in osmotic rigidity (decrease in H50 level, *p* ≤ 4.5 × 10^−5^), while the combined action of RUBI_PHOS increases osmotic fragility (H50: *t*-test *p* ≤ 0.054; corrected Bartlett’s statistic *p* ≤ 0.0151).

#### 3.1.2. OFT: CT Drugs Compromise the Ability of RBCs to Maintain Hydrodynamic Volume

During OFT, we recorded the entire range of scattering angles 0–12°, which made it possible to record MCV, known as the hydrodynamic volume of RBCs, under hypoosmotic loading. The use of laser detection in OFT allowed us to show for the first time that TAX action caused RBC swelling. This happened even with a slight decrease in the osmolality of the medium, meaning that high MCV levels are recorded throughout the 300–100 mOsmol range (Figure 1c). Thus, the MCV_OSM_ of TAX and TAX_PLAT at the osmolality of 200 mOsmol differed from the MCV at 300 mOsmol, 91.6 ± 4.6 vs. 84.6 ± 2.9 (*p* ≤ 0.008). The observed phenomenon of swelling with slightly decreased medium osmolality is considered to be significant for microcirculation and the development of hemolytic anemia. At the same level of buffer osmolality (200 mOsmol), there was no increase in MCV_OSM_ for control, PLAT, and RUBI (Table A1).

#### 3.1.3. OFT: CT Drugs Increase the Heterogeneity of the RBC Population

Due to the fact that the RBC pool consists of cells of different ages, the transformation of their membranes under the influence of CT drugs is not uniform. The index of osmotic heterogeneity W was used as another functional indicator of RBC anisocytosis (Figure 1d). All anticancer drugs were found to cause increased W levels, but TAX (*p* ≤ 9.2 × 10^−8^) and the TAX_PLAT combination (*p* ≤ 1.55 × 10^−6^) contributed the greatest degree of anisocytosis.

#### 3.1.4. OFT: TAX and Its Combinations Disrupts the Discoid Shape of RBCs

**Figure 2:**
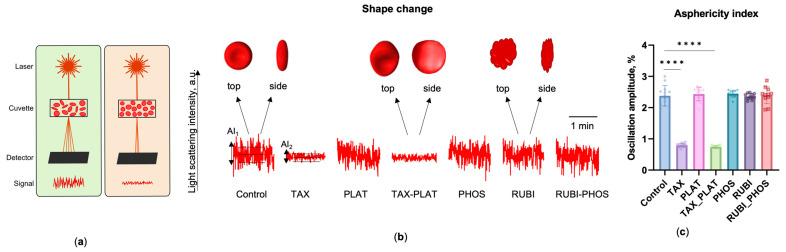
Disruption of native RBC shape under the action of CT drugs obtained by measuring the changes in amplitude of scattering light intensity (asphericity index) on laser diffractometer LaSca-TM. (**a**) At 300 mOsmol, discoid or flattened cells demonstrate a highly oscillated signal (left), while spherical cells demonstrate lower oscillation amplitude (right); (**b**) Representative amplitudes of light-scattering intensity oscillations demonstrate the shape changes of RBCs exposed to CT drugs. Three horizontal lines display the area of registration of the asphericity index (AI_1_, AI_2_). Above the graph, a schematic top and side view of RBCs are shown; (**c**) Asphericity index of RBCs, indicating TAX and its combination led to cell spherization. Data are presented as mean ± SD, *n* = 15 donors, one way ANOVA, Tukey HSD post-hoc; ****, *p* ≤ 0.0001 compared to control.

Apart from recording cells stiffness/fragility, volume, and heterogeneity in mechanical characteristics, OFT based on laser diffractometry allows us to assess cell sphericity. Normal RBCs are diskocytes, so a signal of different amplitude (oscillations) is recorded during stochastic cell rotation in front of the detector during the measurement (Figure 2a). The amplitude of the signal from spherical particles decreases sharply, allowing this parameter to be used to estimate the shape of the RBCs (Figure 2a,b). To describe the RBCs’ shape change, we introduced the asphericity index, which is the amplitude of the SLI oscillations normalised to the average SLI of the sample at the registration angle of 2.5°.

The results show that TAX and TAX_PLAT reduced the asphericity index on average by three-fold (Figure 2c). Under the action of PLAT, PHOS, and RUBI, the flattened shape of RBCs did not change, and the amplitude of oscillations did not differ from controls (*p* ≤ 0.96). We state “flattened” rather than “discoidal” because it could be other variants such as echinocytes. Changes in the asphericity index values were associated with impaired ability of RBCs to maintain their volume after incubation with TAX and TAX_PLAT, which was recorded by OFT (Figure 1c).

Summarizing, the results of OFT showed that the action of TAX and the combined action of TAX_PLAT led to a significant transformation of RBC membranes. Part of the cell population swelled and lysed rapidly, whereas the remaining portion of the cells showed increased resistance (rigidity) to the osmotic load. Incubation with TAX led to spherization of RBCs. The combined action of RUBI_PHOS, but not the individual drugs, contributed to the osmotic fragility of RBCs.

### 3.2. CT Drugs Change the Hematological and Morphometric Characteristics of RBCs

#### 3.2.1. Hematological Analysis: CT Drugs Cause Increased Cell Volume and Population Heterogeneity

**Figure 3:**
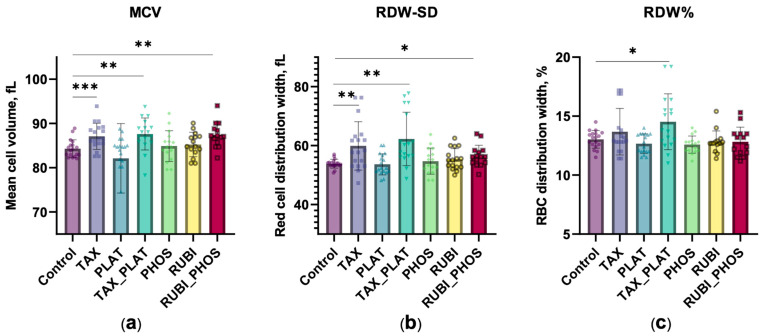
The action of CT drugs impaired the volume regulation of RBCs, resulting in increased MCV and volume heterogeneity. (**a**) MCV, (**b**) RDW-SD, and (**c**) RDW% calculated by Medonic-M hematology analyser. Data are presented as mean ± SD, *n* = 15 donors, one way ANOVA, Tukey HSD post-hoc; *, *p* ≤ 0.05, **, *p* ≤ 0.01, ***, *p* ≤ 0.001 compared to control.

In addition to OFT_,_ standard clinically approved hematological tests were performed. They showed that incubation of RBCs with TAX and TAX_PLAT resulted in a significant increase in mean corpuscular volume (MCV) levels (Figure 3a). For PHOS and RUBI, MCV levels did not differ from control. However, the MCV level was significantly higher when the cells were treated with their combination compared to the untreated control cells (*p* ≤ 0.0047).

The cytological response may vary significantly within the same population during exposure to xenobiotics. Changes in RBC volume under the action of CT drugs did not occur in a uniform manner within the cell pool, which was recorded as an increase in red blood cell distribution width (RDW). In Figure 3b, we show RDW-SD, an index that is included in the routine blood count. In contrast to the more commonly used RDW% (Figure 3c), which calculates the coefficient of variation of the RBC’s volume normalised by the average cell volume, RDW-SD shows a direct measurement of the width of the MCV histogram at 20% of its height. The results showed that RDW-SD was more sensitive to external factors than RDW% (Figure 3b,c): RDW-SD was significantly higher under the action of TAX, TAX_PLAT, and RUBI_PHOS, while RDW% revealed the differences only between control and TAX_PLAT. We believe that RDW-SD may be more useful in the individual assessment of anisocytosis in cancer patients as a more sensitive parameter for minor changes in RBC volume.

#### 3.2.2. Confocal Microscopy: CT Drugs Change the Morphology of RBCs

**Figure 4:**
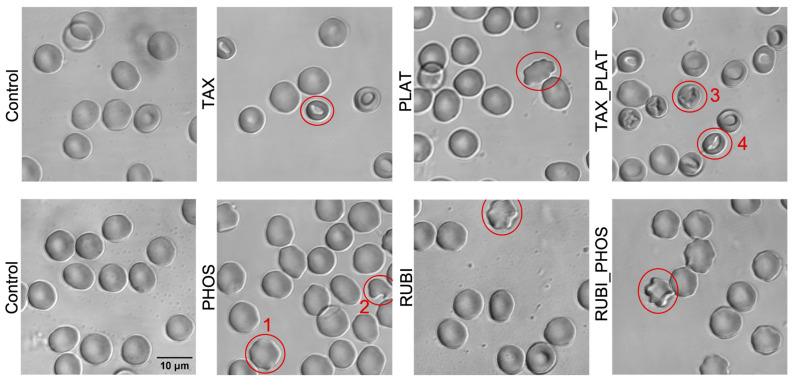
Disturbance of erythrocyte morphology under the action of CT drugs. Representative pseudocoloured confocal images (Leica TCS SP5 MP) of non-fixed RBCs show the appearance of anisocytosis and the disruption of RBCs’ native morphology (red circled RBCs as an example): control cells had the normal shape of discocytes, TAX—discocytes and stomatocytes I–II, PLAT—discocytes and echinocytes I, PHOS—discocytes, echinocytes I (1) and schistocytes (2), RUBI—discocytes and echinocytes I; the combinations of drugs intensified the change in morphology of RBCs: TAX_PLAT demonstrated discocytes and stomatocytes at different stages (3, 4) while RUBI_PHOS—discocytes and echinocytes II.

To investigate the disorders detected during the OFT in more detail and to clarify the data of RBC shape changes, we performed 4 visualization experiments of nonfixed cells performed with confocal microscopy. Image analysis showed that all drugs influenced RBC morphology (Figure 4). Incubation of RBCs with TAX caused stomatocytosis, which is consistent with other studies [25,47]. The action of PLAT was gentler but led to the appearance of echinocytes I. Co-incubation with TAX_PLAT caused pronounced poikilocytosis—the appearance of stomatocytes III–IV. RBCs exposed to PHOS and RUBI mostly retained normal morphology. Nevertheless, it was noted that PHOS, RUBI, and the combination RUBI_PHOS caused the appearance of echinocytes I, eliptocytes, and a single appearance of shistocytes (fragmented RBCs). The classification of RBC forms is given according to [24,48].

**Figure A1:**
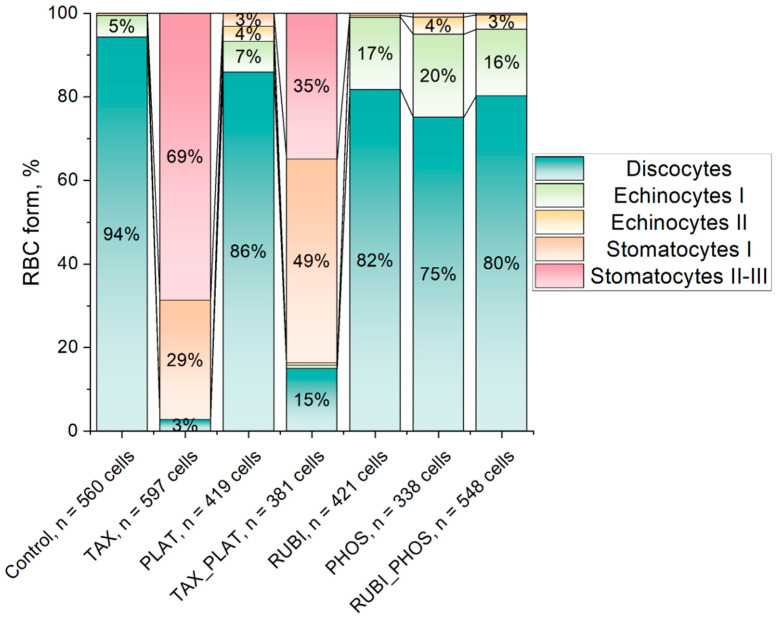
Statistics of RBCs forms according to the confocal microscopy images obtained after the incubation with CT drugs.

Confocal imaging showed that contact of RBCs with TAX and TAX_PLAT caused stomatocytosis, while contact with PLAT, PHOS, RUBI, and RUBI_PHOS caused predominantly echinocytosis. The full statistics of RBC forms are presented in Figure A1. It should be emphasised that echinocytosis is a reversal process [49,50], whereas stomatocytosis is definitely a pathology.

### 3.3. Flow Cytometry: Assessment of Membrane Transformation and Viability of RBCs

Natural aging or induced accelerated RBC death is accompanied by physicochemical changes aimed to introduce recognition markers for macrophages. Such markers are the externalization of phosphatidylserine (PS), expression of neoantigens on the membrane surface, and impaired deformability [51]. We used two standard tests for RBC membrane transformation analysis to assess the degree of damage and readiness of RBCs for removal from microcirculation [52]. The first one is the PS binding to Annexin V, which characterises the relatively early stages of membrane transformation. The second one is a band3 clustering test with EMA labeling, characterising the final stages of RBCs’ transformation before clearance. Additionally, we measured integral cell viability using the Calcein-AM test, which shows the activity of intracellular esterases.

#### 3.3.1. Annexin-Positive Cells Test

**Figure 5:**
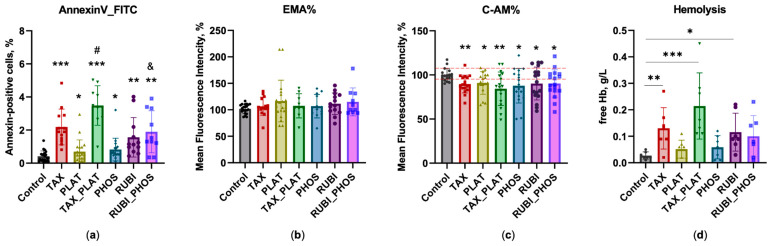
Effects of the anticancer drugs on RBCs’ cytology parameters. (**a**) Annexin V test: all drugs induced the externalization of PS in RBCs (*n* = 10 donors); (**b**) EMA test revealed no membrane changes associated with Band3 translocation (*n* = 10 donors); (**c**) Calcein-AM test: CT drugs reduced RBCs’ vitality. RUBI vs. control *p* = 0.052 (*n* = 13 donors); (**d**) Free Hb content in RBCs’ incubation medium (*n* = 7 donors). Data are presented as mean ± SD, one way ANOVA, Tukey HSD post-hoc (C-AM, EMA, Hemolysis) and Dunn’s post-hoc (AnnexinV); *, *p* ≤ 0.05, **, *p* ≤ 0.01, ***, *p* ≤ 0.001 compared to control; #, *p* = 0.0128, compared to TAX, &, *p* ≤ 0.05, compared to PHOS.

According to the Annexin V test, the action of CT drugs on RBCs caused lipid asymmetry disruption, which was expressed in the phosphatidylserine exit to the outer side of the membrane (Figure 5a). The number of annexin-positive RBCs was significantly higher than in control for TAX (*p* ≤ 0.001) and TAX_PLAT (*p* ≤ 0.04). All the other investigated drugs also showed an increased number of annexin-positive cells.

#### 3.3.2. EMA Test: Action of Anticancer Drugs Does Not Cause Clustering of Band3 Membrane Complexes

Old or damaged RBCs are removed by phagocytosis in the spleen sinuses, as well as in the liver and bone marrow [53]. Macrophages recognize the erythrocytes to be removed by a number of aging markers, which include, among others, the exposure of new binding sites on the membrane surface. Since RBCs do not have the ability to synthesize new proteins, the creation of these binding sites occurs through the transformation of the existing membrane complexes with the band3 protein. These complexes form the core of the macrocomplexes of integrin and peripheral membrane proteins [54]. Profound changes in the structure of RBCs’ membranes include the exposure of CD47 phagocytosis inhibitor sites located in the band3 complex, which switch them to the “eat me” signal of old/injured RBCs [55]. EMA dye binds predominantly with band3 protein. Increased intensity of EMA-dyed cells signifies the transformation of band3 complexes, which accompany the final stages of a RBC’s life.

After the incubation of RBCs with CT drugs, no significant disturbances of band3 transmembrane complexes were detected (Figure 5b). Only a slight increase in MFI values for RUBI_PHOS (*p* ≤ 0.054) was noted. The negative result obtained may be related to insufficient time for the deep transformation of the membranes. Previously, we found [38] that a significant increase in MFI while staining RBCs exposed to tert-butyl hydroperoxide requires at least 3 h of incubation with relatively high oxidant concentrations. Maximum MFI levels of band3 clusters were observed after 24 h exposure.

**Figure A2:**
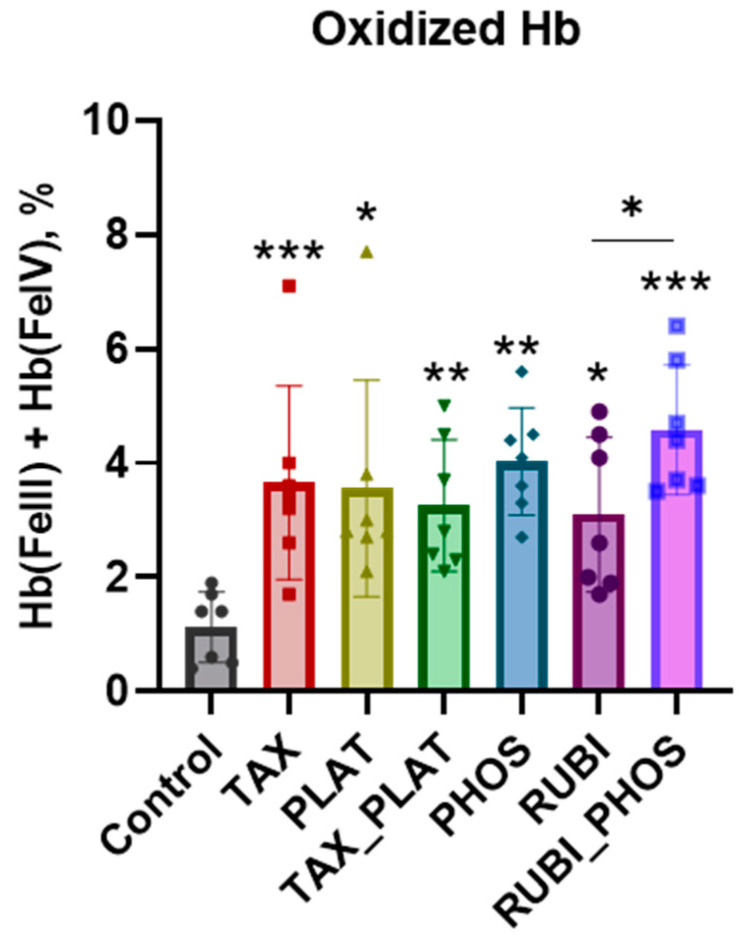
The action of CT drugs has contributed to the accumulation of oxidised forms of hemoglobin: the formation of reversible metHb and non reversible hemichrome. Spectral scans of Hb from hypoosmotically lysed RBCs after 3 h treatment with the CT drugs showed the elevated levels of oxidised Hb. Spectral range was from 450 to 700 nm. Data are presented as Mean ± SD, *n* = 7; one way ANOVA, Tukey HSD post hoc, *, *p* ≤ 0.05, **, *p* ≤ 0.01, ***, *p* ≤ 0.001 compared to control.

For a deeper analysis of the transformation of the RBC membrane and cytoskeleton under the influence of chemotherapy drugs, we investigated the hemichrome and methemoglobin (metHb) content in our samples. Hemichromes trigger prolonged band3 phosphorylation, which causes disruption of interactions between the cytoskeleton and membrane protein, which in turn contributes to shape disruption and enhances hemolysis [56]. MetHb leads to increased lateral diffusion of band3 complexes and cluster formation due to its high-affinity binding to the cytoplasmic band3 domain [52]. Spectroscopy of lysed RBCs pre-incubated with CT drugs showed that after 3–4 h of exposure there is an increase in the formation of reversible metHb (Hb(FeIII)) and nonreversible hemichrome (Hb(FeIV)) (Figure A2), which may also contribute to anemia during chemotherapy. Since RBC transformation, deformability, and band3 clustering strongly depends on oxidant concentration/time and the ratio of oxidant to cell count, we can conclude that the level of formation of oxidised forms of hemoglobin was not sufficient for the deep membrane transformation that is observed under severe oxidative stress.

#### 3.3.3. Calcein-AM Vitality Test

Most of the intracellular esterases can hydrolyze a wide range of substrates [45], such as nonfluorescent calcein-AM, converting it into fluorescent calcein. Thus, the decreased MFI in the calcein-AM test reflects a complex disruption of the enzyme activity that supports the cell’s vitality. This test is used as a quick option to assess the viability of nucleus-free cells [38]. Apart from RUBI (*p* ≤ 0.0502), all the CT drugs and their combinations caused a decrease in the activity of intracellular esterases (Figure 5c), which is considered as a decrease in overall cell viability.

A significant decrease in MFI under the action of TAX (*p* ≤ 0.0079) and TAX_PLAT (*p* ≤ 0.0013) may be associated not only with inhibition of esterase activity but also with dye wash-out from cells when the integrity of membranes is disrupted. The analysis of free hemoglobin content (*n* = 7 donors) showed a significant increase in hemolysis of RBCs (Figure 5d). So, the decrease of calcein MFI for TAX- and TAX_PLAT-treated RBCs may be connected with both a decrease in intracellular esterase activity and a disruption of membrane integrity.

**Figure A3:**
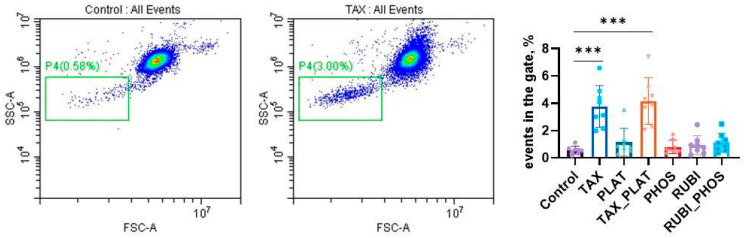
CT drugs induced RBCs transformation and microparticle formation. Representative SSC/FSC dot plots of control RBCs and RBCs under the action of TAX. The data shows the microparticle formation (left, green gate). The statistics of the formation of microparticles by CT drugs is shown on the right panel. Template and gating are corresponded to the left scan. Data are presented as Mean ± SD, *n* = 7 donors; one way ANOVA, Tukey HSD post hoc, ***, *p* ≤ 0.001 compared to control.

In addition, we should note that the analysis of cell distribution in FSC-SSC coordinates showed that TAX treatment stimulated microparticle formation in the samples, which was recorded by the accumulation of events in the gate calibrated on latex beads 3 µm in diameter (Figure A3).

In summary, the action of CT drugs leads to a decrease in the activity of intracellular esterases and the externalization of phosphatidylserine in RBCs. The most severe transformation of the cells occurred under the action of TAX: formation of microparticles and disruption of membrane integrity with the release of free hemoglobin into the incubation medium occurred.

### 3.4. Microfluidic Analysis: CT Drugs Degrade RBCs’ Ability to Pass through the Microchannels

As an integrative test of the damage received by erythrocytes during incubation with CT drugs, we performed a microfluidic analysis of cell transport in 2.5 × 8 × 200 μm microchannels [31,41]. The microfluidic analysis is a single-cell high-throughput method (100–1000 cells in one experiment) that can estimate cell functionality under microcirculation conditions.

**Figure 6:**
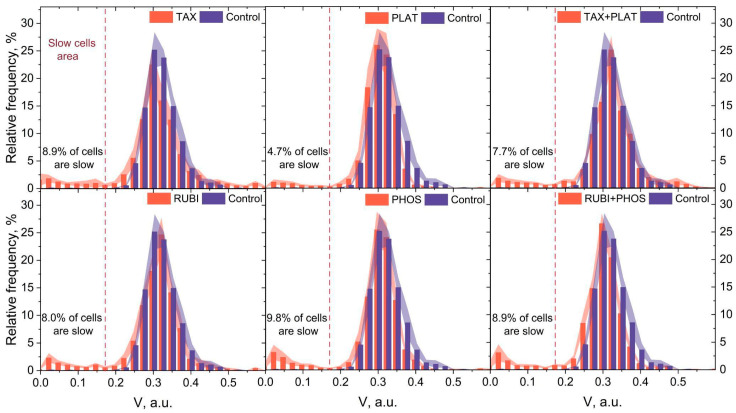
Distributions of RBCs’ relative velocities in the microchannels of the microfluidic device. The action of CT drugs did not cause a significant shift in the main peak of cells’ velocities but led to the appearance of a subpopulation of slow cells for all used chemotherapy drugs. The data are presented as mean ± SE, and the error bars are shaded. *n* = 10 donors.

All circulating RBCs have different ages; therefore, velocity distribution of untreated cells moving in the microchannels (Figure 6, blue histogram) can be approximated with a Gaussian function. Previously it was shown that when the cells are under oxidative stress, their population splits into two subpopulations of normal, undamaged cells and slow-damaged cells according to the capability of their built-in antioxidant defense system [31]. This leads to changing the velocity distribution from single modal to bimodal. For RBCs treated with CT drugs, similar to oxidative stress conditions, the velocity histogram transforms into bi- or trimodal (Figure 6, orange histograms).

The distribution changes can be separated into two variants. The first one is the appearance of the subpopulation of slow cells, which is typical for all investigated drugs (Figure 6, areas on the left of the graphs). These slow cells are unable to maintain the optimal speed in the microchannels inherent to the control cells due to impaired volumetric regulation and/or due to decreased deformation capacity. The second one is the appearance of fast cells (found only for TAX and TAX_PLAT) associated with the formation of microparticles (Figure A3). It is important to note that both transit abnormalities indicate pathology in the RBC population: decreased transit capacity carries the risk of microvascular blockage, while its increase leads to insufficient time for erythrocytes to be near the endothelial cells for efficient gas exchange.

The cutoff for counting slow cells was assumed to be 0.175 a.u. (Figure 6, dashed red line) resulting from the fact that the region below these values accounts for less than 0.45 ± 0.20% of control cells. Figure 6 displays the relative number of slow RBCs that had impaired microchannel transit characteristics. Although the appearance of the slow cell subpopulation was recorded for all CT drugs used (Control 0.45 ± 0.20%, TAX 8.9% ± 3.29%, PLAT 4.7% ± 1.88%, TAX_PLAT 7.7% ± 2.39%, RUBI 8.0% ± 2. 10%, PHOS 9.8% ± 3.76%, RUBI_PHOS 8.9% ± 3.30%), the data analysis showed no statistically significant differences between the number of cells in these subpopulations when different CT drugs were used. We attribute this to the fact that microfluidic analysis data are an integral indicator of the above-mentioned disorders in CT-drug-treated RBCs. At the same time, the main part of the population of RBCs under the action of CT drugs moved with a velocity inherent to the control cells: the position of the main velocity peak of the distribution histograms had no significant changes compared to the control cells.

**Figure A4:**
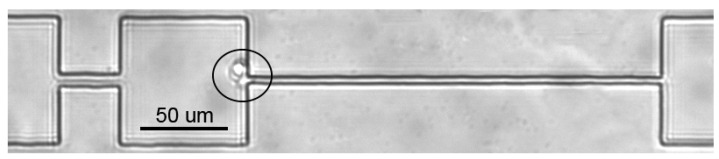
Representative image of a microchannel occlusion by RBCs treated with TAX drug.

**Figure A5:**
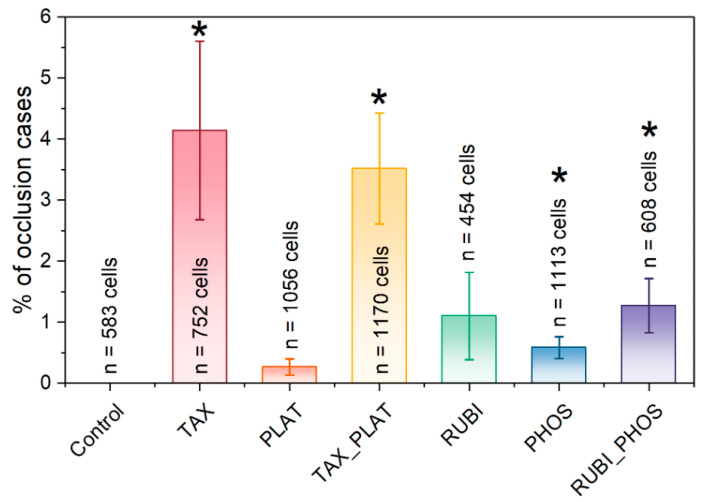
Percentage of microchannels occlusion cases. The number of cases of microchannel occlusions was counted manually; an occlusion was recorded if erythrocyte retention was at least 10 s. Data presented as mean ± SE, n = 3 donors, 2-tailed non-paired *t*-test; *, *p* ≤ 0.05 compared to control.

Experimental results also demonstrated increased cell adhesion to the microchannels walls and to each other. Together with an increase in cell volume, shape changes, and changes in deformational properties under TAX, TAX_PLAT, PHOS, and RUBI_PHOS, this resulted in microchannels occlusions (Figure A4 and Figure A5).

To summarize, all chemotherapy drugs had a damaging effect on the ability of several RBCs (4.7–9.8%) to pass microchannels. Moreover, it is important to note that the action of TAX and TAX_PLAT on RBCs resulted in a high number of microchannel occlusions.

## 4. Discussion

The aim of the study is to evaluate the disorders of RBCs exposed to CT drugs. We analysed the effects of the four main pharmacological compositions of CT drugs and their combinations included in current treatment regimens for solid tumours. The drugs differed in their action mechanism, and, as expected, TAX and its combination with PLAT had the most aggressive effect on RBCs. Such a drug combination decreased RBCs’ total viability (Figure 5c), caused vesiculation (Figure A3), stomatocytosis (Figure 4 and Figure A1 [24,25,47]), and hemolysis (Figure 5d) and led to cells’ swelling (Figure 3a).

The main mechanism of TAX therapeutic action is related to disturbance of tubulin microtubules polymerization, which halts entry to anaphase-preventing cell division [3]. In the early stages of maturation, microtubules are present in pro-erythroblasts, but during maturation tubulin shifts to a disorganised structure. It continues to play a structural role in the RBC sedimentable fraction [57]. However, in RBCs there is no tubulin, but there is another class of cytoskeleton structures—actin microfilaments, on which TAX can also act. Its action on the membrane structures of RBCs leads to an increase in the transverse bonds between actin subunits and other proteins, such as tropomyosin, protein 4.1R, and dematin, which through adducin are connected with band3 membrane proteins [11,58]. Therefore, actin transformation, caused by TAX, affects membrane structural components and their links with the cell’s cytoskeleton, which was indirectly shown with the Annexin V test (Figure 5a). This leads to violations of RBC shape and volume, which we observed in the osmotic fragility test (Figure 1c and Figure 2c), hematological analysis (Figure 3a), and confocal microscopy (Figure 4 and Figure A1). Hypothetically, TAX may also interfere with the molecular apparatus responsible for the perception and regulation of plasma membrane curvature, the nonmuscle myosin II and actin complex. Additionally, through actin polymerization TAX can distort signal transduction from transmembrane mechanosensors to cell volume regulation mechanisms [59].

In clinical treatment, TAX is usually used with platinum-based drugs. Our data show that PLAT itself has a low toxicity effect on RBCs because its main target in cancer cells is DNA. Therefore, in its combination with TAX, cell transformation mainly occurred due to TAX action. This follows from the close values of all measured parameters of cells treated with TAX and TAX_PLAT. Additionally, it should be mentioned that comparing our data with previously published results we can say that carboplatin has a much lower influence on RBCs than the older platinum-based drug cisplatin [18,19,60,61]. This might be due to the lower affinity of carboplatin for proteins [62]. Thus, carboplatin is preferred for therapy for preventing anemia.

Unlike taxanes, whose target is the cell cytoskeleton, the influence of anthracycline drugs (RUBI) did not lead to strong changes in RBCs’ shape and volume. It is of note, that one of the targets of RUBI in cancer cells along with DNA is the lipid part of the membrane [7]. Therefore, we saw membrane transformations manifested in the higher Annexin V signal and hemolysis rate (Figure 5a,d). Nevertheless, we did not observe large changes in cell volume and shape (Figure 3a). Confocal microscopy revealed the appearance of the first order echinocytes, which is a reversible violation (Figure 4 and Figure A1). The evidence is insufficient to link these disruptions of RBCs that we have identified to any mechanism of membrane transformation under RUBI action. The only conclusion possible is that RUBI interacts with RBC membrane structures, and its effects could be enhanced by combination with PHOS. By itself, PHOS has a low toxicity effect due to the fact that it should be transformed in cells into active forms by intracellular phosphatases. Upon such transformation, its main target is DNA, which is absent in RBCs.

Microfluidic functional analysis of CT-treated RBCs demonstrated the appearance of a subpopulation of slow-damaged cells (Figure 6) with a lower ability to move in microcapillaries than the healthy ones. Accordingly, these cells are removed from the blood and destroyed in the spleen. However, the number of slow cells did not exceed 10% for all investigated drugs and their combinations. Still, it should be noted that even this small number of cells with an altered biophysical phenotype could lead to profound consequences for blood rheology in cancer patients [63].

Despite the significant differences in the cytological and morphological status of RBCs treated with different drugs, the number of slow cells was approximately equal in all groups. We hypothesize that this uniform response is related to the application of all the drugs in therapeutic concentrations, which provides a balance between drugs’ antiproliferative activities and cytotoxic effects. Additionally, the treatment of RBCs with TAX, TAX_PLAT, and RUBI_PHOS drugs resulted in occlusion of the microchannels due to cell adhesion to the walls and to each other. This effect might also lead to violations in microcirculation and anemia.

Interpretation of the obtained results should be performed keeping in mind several limitations. In vitro conditions during experimental studies were quite different from in vivo conditions in cancer patients. To evaluate the effects of direct exposure to pharmaceuticals drugs on human RBCs in vitro, washed cells were used. This approach revealed the negative effects of CT drugs, but it cannot be equivalent to the response of RBCs to these drugs in vivo. The response in patients may be more complex and depend on the condition of the red cell pool (impaired erythropoiesis and abnormal erythrocyte survival). Additionally, blood plasma composition is significantly different from the HEPES buffer used in the study. Many CT drugs in blood circulation can bind to plasma proteins, such as albumin, which may protect RBCs from direct cytotoxic injury. However, it is possible that the degree of negative effects of CT drugs will accumulate with several cycles of chemotherapy.

## 5. Conclusions

Our work shows that the response of RBCs to the direct action of commercial pharmaceutical CT drugs (impaired volume regulation, reduced cell vitality, shape change, and imbalance resistance to osmotic loading) depended upon the pharmacological group of the drugs. Drug combinations had additive effects. The greatest cytonegative effect was that of paclitaxel as an agent targeting cytoskeleton proteins. Drugs whose main target is DNA showed significantly lower toxicity. Microfluidic analysis showed that despite the differences in the level of cytological abnormalities, the microrheological behavior of erythrocytes was similar to and independent of the pharmacology of the drug. This can be attributed to the action of the drugs within therapeutic concentrations, which provides the balance between cytotoxicity in tumour and healthy cells.

## Data Availability

The data presented in this study are available on request from the corresponding author.

## Appendix B

**Table A1:** The hematology, OFT, spectrophotometry and microfluidics indicators of RBCs treated by the CT drugs; one way ANOVA, post hoc Dunnett’s multiple comparisons test with adjusted *p* value. *p* ≤ compared to corresponding control.

	Control	TAX	PLAT	TAX_PLAT	PHOS	RUBI	RUBI_PHOS
Hematology analysis							
MCV, fL *n* = 15	83.8 ± 3.1	87.1 ± 2.9 *p* ≤ 0.0321	82.7 ± 7.6 *p ≤* 0.416	87.0 ± 4.7 *p* ≤ 0.0106	84.9 ± 3.5 *p* ≤ 0.326	85.2 ± 2.8 *p* ≤ 0.156	86.7 ± 2.3 *p* ≤ 0.0345
RDW-SD, fL *n* = 15	53.9 ± 1.5	59.9 ± 8.2 *p* ≤ 0.007	53.7 ± 3.6 *p* ≤ 0.824	62.3 ± 9.0 *p* ≤ 0.0001	54.7 ± 4.4 *p* ≤ 0.465	55.4 ± 3.6 *p* ≤ 0.140	56.5 ± 3.6 *p* ≤ 0.047
RDW-CV, % *n* = 15	13.0 ± 0.8	13.7 ± 2.0 *p* ≤ 0.208	12.7 ± 2.4 *p* ≤ 0.178	14.5 ± 0.7 *p* ≤ 0.023	12.6 ± 0.9 *p* ≤ 0.090	12.8 ± 0.9 *p* ≤ 0.487	12.9 ± 1.2 *p* ≤ 0.592
Osmotic Fragility Test							
H50, mOsmol *n* = 15	123.9 ± 2.9	119.5 ± 3.9 *p* ≤ 0.001	124.6 ± 4.4 *p* ≤ 0.521	115.1 ± 5.7 *p* ≤ 0.000	124.6 ± 5.1 *p* ≤ 0.610	125.9 ± 3.5 *p* ≤ 0.037	126.1 ± 3.0 *p* ≤ 0.054
W, mOsmol *n* = 15	52.8 ± 1.5	60.2 ± 2.9 *p* ≤ 0.000	54.3 ± 3.7 *p* ≤ 0.341	59.7 ± 3.0 *p* ≤ 0.000	55.7 ± 3.0 *p* ≤ 0.0345	54.7 ± 3.7 *p* ≤ 0.0492	55.2 ± 4.4 *p* ≤ 0.0324
Asphericity Index, a.u. *n* = 15	2.36 ± 0.32	0.79 ± 0.06 *p* ≤ 0.000	2.37 ± 0.33 *p* ≤ 0.967	0.76 ± 0.04 *p* ≤ 0.000	2.39 ± 0.23 *p* ≤ 0.817	2.32 ± 0.17 *p* ≤ 0.628	2.34 ± 0.32 *p* ≤ 0.868
Hemolysis, % 100 mOsmol. *n* = 15	86.5 ± 3.1	72.1 ± 8.3 *p* ≤ 0.000	89.2 ± 5.4 *p* ≤ 0.753	70.0 ± 9.4 *p* ≤ 0.000	88.2 ± 4.7 *p* ≤ 0.938	88.6 ± 4.2 *p* ≤ 0.879	90.6 ± 3.1 *p* ≤ 0.0352
Spectrophotometric analysis							
Free Hb, % *n* = 7	0.03 ± 0.01	0.13 ± 0.08 *p* ≤ 0.0208	0.05 ± 0.03 *p* ≤ 0.09	0.21 ± 0.13 *p* ≤ 0.001	0.06 ± 0.04 *p* ≤ 0.10	0.12 ± 0.07 *p* ≤ 0.02	0.10 ± 0.08 *p* ≤ 0.0493
Microfluidic analysis							
Slow cells, %	0.45 ± 0.20	8.9 ± 3.29	4.7 ± 1.88	7.7 ± 2.39	9.8 ± 3.76	8.0 ± 2.10	8.9 ± 3.30
Number of analysed cells	5386	4379	6389	5188	5010	5420	4936
Occlusions, % *n* = 3	0 ± 0	4.14 ± 1.46 *p* ≤ 0.0472	0.26539 ± 0.13 *p* ≤ 0.1193	3.51702 ± 0.91 *p* ≤ 0.0179	1.10036 ± 0.71 *p* ≤ 0.0305	0.58389 ± 0.18 *p* ≤ 0.1747	1.27221 ± 0.44 *p* ≤ 0.0452
